# Ensuring primary eye care at the school level: a national eye care initiative in Bangladesh

**Published:** 2022-03-01

**Authors:** Nahid Ferdausi, Golam Mostafa

**Affiliations:** 1 Associate Professor: National Institute of Ophthalmology & Hospital, Dhaka, Bangladesh.; 2 Professor and Director: National Institute of Ophthalmology & Hospital, Dhaka, Bangladesh.

**The early detection of possible vision problems through school eye testing is key to ensuring eye health in children.**

## National eye care programme

Bangladesh is a low-income country in South Asia with a population of 160 million, the majority of which (66.5%) lives in rural areas. Despite rapid urbanization and gains in socio-demographic indicators, around 18% of the population (of which 36% is rural and 28%, urban) still lives below the poverty line.^1^ Getting basic eye health care remains a challenge for poor and marginalized sections of the population.

With the objective of improving eye care service delivery at all levels of health care in Bangladesh, the government of Bangladesh has instituted the national eye care (NEC) programme under the 4th Health, Population, and Nutrition Sector Programme (4th HPNSP) for the period 2017–2022. The objective of the operational plan of the NEC is to improve eye care service delivery at all levels of health facilities in Bangladesh. One of the six specific objectives is to control childhood blindness and increase awareness about blindness prevention among the population.^1^

## Vision impairment in children

The age standardized prevalence of blindness is 1.53% in Bangladesh; around 51,200 children are blind; 1.3 million children have refractive errors; and an additional 153,000 children are affected by low vision problems, which are avoidable through timely intervention.^1^ According to a study conducted in a peri-urban setting in Bangladesh, the most commonly observed ocular morbidity was refractive error in children aged less than 15 years.^2^

**Figure:**
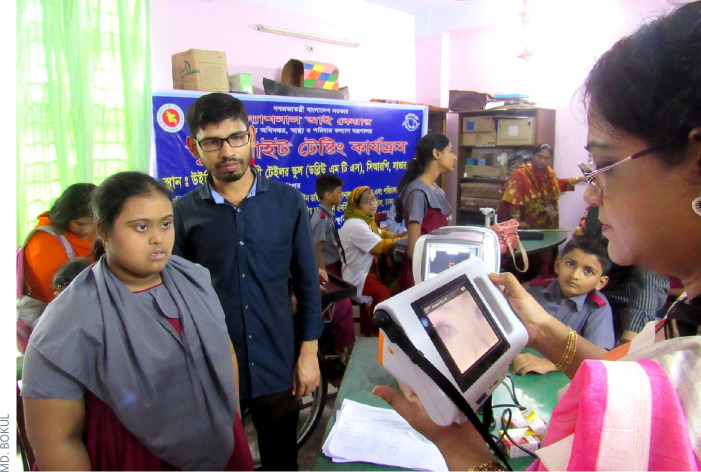
Eye testing camp at an inclusive school in Dhaka. **BANGLADESH**

Since children are not always able to point out vision deficiency at an early enough stage and parents may remain unaware of the gradually developing vision problem, uncorrected refractive errors can have a detrimental impact on children's learning capacity and academic outcomes. Timely eye screening of children helps in the early detection and correction of common eye problems, such as strabismus, developmental cataract, ptosis, amblyopia, and macular dystrophy.

## Ensuring eye health through school sight testing

School health programmes are fully funded by the government of Bangladesh. They have moved from the once narrow practice of a medical examination of children (4 to16 years) to the now broader goal of comprehensive care for the health and wellbeing of children throughout the school years. This change is reflected in the national eye care (NEC) programme. The NEC programme lays special emphasis on the provision of school eye health services through the school sight testing programme (SSTP). All levels of schools, i.e., kindergarten, primary, and secondary, as well as schools for children with disabilities, are included in the NEC programme.

Based on the SSTP findings, the major challenges to providing efficient refractive error services for school children in Bangladesh are: poor health-seeking behaviour; lack of knowledge about refractive status among students, parents, and teachers; poor access to screening; problems with availability and affordability of prescription spectacles; poor compliance of wearing prescribed spectacles; and negative attitudes and beliefs regarding the use of spectacles.

The overall goal of the school sight testing programme is to increase awareness of common eye problems, ensure early detection and proper management of eye problems, provide for correction of refractive errors with appropriate spectacles, and spread awareness of safety measures to prevent ocular injuries.

The programme manager and deputy manager are responsible for the follow-up activities resulting from the programme. The components of the school sight testing programme are:

- *formation of SSTP team*, which would include an ophthalmologist (the team leader), postgraduate students, nurses, and office staff
- *communication* with the school authorities for fixing testing schedules
- *raising awareness* among parents and guardians about child eye care
- *orientation sessions* for school teachers to familiarise them with common eye problems in school-age children and the procedure for vision screening
- *assigning a teacher* as the focal person to communicate with the NEC authorities
- *hands-on training* in vision screening for class teachers
- *vision screening* for students using an E-chart or, in the case of students with autism or cerebral palsy, a vision screener
- *eye examination* with a handheld slit lamp
- *treatment* for eye problems detected (prescribed medicine provided free of cost)
- *referral* to a higher level of eye care centre, if needed (e.g., for cycloplegic refraction)
- *subjective refraction* conducted after objective refraction (the latter done with auto-refractometer and retinoscope) for prescribing spectacles
- *provision of prescribed spectacles* to children in school, free of cost, within seven to ten days from an optical shop assigned by the NEC.

Under the school sight testing programme, teachers are given primary training by the government. To make the school sight testing programme cost-effective, visual acuity of less than 6/9 is taken to be defective vision. It is advised that students whose vision is 6/9 have their vision checked again six months later by a class teacher. If visual acuity is less than 6/9, the pinhole test is carried out. If vision can be corrected after testing using the pinhole test, objective refraction and subjective refraction are performed for prescription of spectacles. If vision does not improve, the child is referred to an eye hospital for further investigations and treatment.

Under this programme, on average, 1,650 children are screened each year. In past 12 months, 81 children have been provided with spectacles, and 25 children were referred to nearby government hospitals.

Raising awareness among teachers, parents, and children about eye health and ensuring that all school children be offered visual acuity screening are critical to the early detection of possible vision problems.

**Figure:**
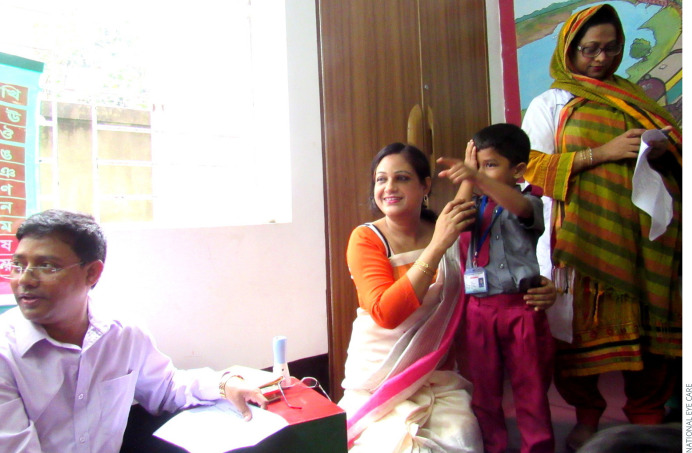
A child undergoing vision screening. **BANGLADESH**
